# Color me khmao: the effects of social factors on colorism among Khmer women

**DOI:** 10.3389/fsoc.2025.1499198

**Published:** 2025-02-17

**Authors:** Vanessa Lakana Veak

**Affiliations:** Department of Sociology, Stanford University, Stanford, CA, United States

**Keywords:** colorism, Cambodia, Khmer women, family, self-esteem and belonging, wellbeing, community, skin tone discrimination

## Abstract

**Introduction:**

Given the lack of academic literature on colorism within the Cambodian community and the lack of focus on how colorism influences experiences in this context, this study seeks to analyze how Khmer women’s perceptions of colorism are shaped by their family and community environments.

**Methods:**

The data and methods consist of 40 in-depth qualitative interviews with Khmer women, primarily those living in the United States and Cambodia, with their experiences of colorism analyzed through thematic analysis.

**Results:**

Findings reveal that factors such as family support and cultural connections to Khmer identity interact with relative skin tone within families and broader communities to shape self-esteem and experiences with colorism. Women with darker skin did not necessarily have the lowest self-esteem, especially when they had supportive families and lived in communities where darker skin tones were more prevalent. Conversely, women with lighter skin did not necessarily have the highest self-esteem, especially when their families reinforced color hierarchies and they lived in communities dominated by White or East Asian Americans.

**Discussion:**

By further examining this structural issue, colorism, the study highlights how communities of color can work toward racial and ethnic justice while developing strategies for future generations to challenge and move beyond colorism.

## 1 Introduction

[Growing up,] my brother-in-law called me the N-word. He’ll just call me, “‘Ey little N-word! Hey little N-word!” and I was the one that was dubbed me khmao/មិខ្មៅ (black, derogatory nickname) … I mean I’ve known a lot of me khmaos growing up. There was always one in the family. I do not know if it’s derogatory or not, but there were times when if I was mean, they were like, “ម៉ោពីវាខ្មៅអញ្ចឹងបានវាកាចដូចអញ្ចឹង ឬកខ្មៅសាច់ខ្មៅអាក្រក់ដល់ហើយ” (it’s because she’s black, that’s why she’s angry like that. Black attitude and skin black, so ugly).

Regardless of Khmer language fluency, the word khmao/ខ្មៅ, meaning black, holds familiarity to the vast majority of the Cambodian community, though not as a literal color but primarily in regards to skin complexion. Soriya, a 37-year-old 1.5-generation Khmer American in Minnesota, shares her experience in the quote above growing up with the nickname me khmao, a commonly shared nickname across Cambodians and colorism. Colorism is defined as the structural occurrence of white or light-skin supremacy associated with status, privilege, and other positive stereotypes, while darker skin is perceived with disdain and other negative stereotypes (Dixon and Telles, 2017). For my study, I use skin tone and complexion interchangeably, and I specify *White* as the racial group or of European descent to distinguish it from *white* as pertaining to skin color, similar to *Black* and *black*. In addition, I extend this definition to involve the discrimination and preference for certain facial features and hair texture, as the majority of interviewees in my study discussed feeling self-conscious about their textured hair and flatter noses in conjunction with their skin color, associating lighter-skinned Asians or the Asian standard of beauty with having slimmer noses and silky and straight hair (Rondilla and Spickard, 2007). While research on colorism has been increasing recently over the decades, the concept of colorism has existed in all regions and racial groups for centuries (Dixon and Telles, 2017). Because of the United States’ color line shifting from majority White people to majority people of color given the rise of Asian, Black, and Latinx immigrants and multiracial populations, colorism becomes more prominent as racial distinctions may become increasingly blurred, hence the rise in literature (Burton et al., 2010).

Despite increased attention to colorism overall and especially in the U.S., there is a lack of research on Southeast Asian and Southeast Asian American communities. Thus, my study focuses on the effects of colorism among Khmer women, who are darker-skinned on average relative to Asia’s ideal skin tone, and among East Asians and East Asian Americans. I focus on women, particularly due to the greater extent to which they are affected by colorism in the context of marriage, value, femininity, and other social capital (Glenn, 2008). For women, lightness is associated with femininity, beauty, and purity; lightness brings women perceived value in the marriage market and social opportunities (Hunter, 2021; Rondilla and Spickard, 2007). This study aims to demonstrate how Khmer women, who are also marginalized by indigenous erasure, are vulnerable to the harmful and systemic effects of colorism.

Existing literature suggests that colorism feels more overt and culturally emphasized in Asia than in the United States (Khanna, 2020). In addition to this, colorism in Asia existed prior to European contact and thus is not entirely rooted in seeking European Whiteness (Dixon and Telles, 2017). While in the Philippines, Spanish and the United States colonialism majorly contributed to a color hierarchy with lighter skin at the top (Rondilla, 2012); lighter skin was historically a sign of the upper class in East Asian countries like China, Japan, and Korea, with the ideal skin color described as snow (Yeung, 2016; Glenn, 2008); and while British colonialism exacerbated colorism in South Asia, the caste system existing far prior also contributed to stratification based on skin color (Modi, 2016). With a variety of historical contexts in Asia, further research on colorism across Asian communities is imperative.

I focus on Khmer American women additionally, as opposed to solely Cambodian nationals, due to the pressure that arises from being in a White-dominated society and appearing stereotypically Asian through a White American lens (Trieu and Lee, 2018). The observation of colorism in a comparative national context indicates the influence of community dynamics and the intergenerational transmission of colorism within families. Although some colorism scholarship addresses the relationship between family and colorism (Tharps, 2017) and interpersonal outcomes such as job and social opportunities (Monk, 2014), there is a lack of literature on the impact of the overall complexion of community on perceptions of colorism and its factor into self-esteem and how one views their skin color. This study seeks to fill this missing gap, bringing the question of how Khmer women’s perceptions of colorism are shaped by their family and community contexts.

My findings suggest that Cambodians of all generations acknowledge the belief that lighter skin will equate to better social and economic opportunities, a large number desiring to possess lighter skin, straighter hair, and a more slender nose themselves at some point in their lives, identical to several colorism research findings with other communities (Mathews and Johnson, 2015; Norwood, 2015; Rondilla, 2012). Although all respondents described experiences with colorism, the extent to which their skin color was associated with self-esteem varied by four key factors: (a) Family Support, (b) Skin Tone in the Family, (c) Community Color; and (d) Connection to Khmer Culture. I posit that possessing at least two of these factors that contribute to lower self-esteem will thus generally lead to lower self-esteem and a negative view of one’s skin color. Similarly, possessing at least two of these factors contributes to a higher self-esteem, which will thus generally lead to a higher self-esteem and positive view of one’s skin color. As a result, these family and community contexts operate collectively rather than one theme dominating over another, illustrating the intricate dynamics between family, community, and self-esteem. Additionally, due to observing colorism as a structural issue in the Cambodian community, predominantly in the United States and Cambodia, participants begin considering strategies for future generations to navigate and combat colorism.

### 1.1 The Khmer ethnic identity

Cambodia is a multiethnic country, with the ethnic Khmer group making up about 90% of the country’s total population, and ethnic Chinese, Vietnamese, Cham, and mountain-dwelling ethnic groups accounting for the remainder 5 to 13% (National Institute of Statistics, 2020; Hein, 2006). As intermarriage and mixing across different groups in Cambodia increase, the blurred ethnic lines and skin tone variation increase, in turn, parallel to the United States. Although a small minority, Chinese Cambodians have currently been the most dominant group economically, significantly controlling Cambodia’s commerce and businesses, leading to higher-paying and higher-position occupations, creating conflict among other Cambodians (Hein, 2006; Willmott, 1981), and exemplifying wealthy, lighter-skinned Asians in Cambodia. The indigenous Khmer ethnic group, apart from being the majority of Cambodia’s population, is, on average, darker in skin tone in comparison to the Chinese ethnic group and East Asians in general. Prasso (1995) asserts that there is a preference for lighter complexions in Cambodians. In addition, due to Cambodia’s unique history with the atrocities of the Khmer Rouge regime, many Cambodians experience some desire to distance themselves from the Cambodian identity (Chan, 2004; Kinzie, 1985). Given all of these factors, Khmer people are more susceptible to colorism, especially with a social hierarchy that places the darker-skinned Khmer people at the bottom and lighter-skinned Asians at the top in not only East and Southeast Asia but within Cambodia as well.

As of 2020, with a population of approximately 339,000 in the United States (Budiman, 2021), Cambodian Americans have 38% limited English proficiency compared to 9% of the total population, are the Southeast Asian American group least likely to have completed high school, 23% low-income, and 20% in poverty, and suffer from a higher incidence of severe mental health problems than any other group and the general population overall (Southeast Asia Resource Action Center, 2020a; Southeast Asia Resource Action Center, 2020b). After 1985, many Cambodians joined gangs due to the treatment they received from Americans, such as getting jumped or alienated for being Asian, and to cope and compensate for their lack of attention and treatment from teachers, peers, and parents, as well as a form of affirming their Khmerness or Cambodianness by having pride for their gang of Cambodians (Needham and Quintiliani, 2007). This may also have contributed to the association of Cambodians with gang life, as well as the media painting a negative light on Cambodian youth when gang warfare became the most violent between the 1980s and 1990s despite the majority of Cambodian youth doing well in school (Chan, 2004).

As a result of the systemic and social conditions that Cambodian Americans possess–more often darker-skinned Khmer Americans–society perceives them with negative stereotypes such as unintelligent, gang-affiliated, poor, and uncivilized (Chhuon, 2014), similar to darker-skinned Black Americans (Hannon et al., 2013; Welch, 2007). In contrast, for both Khmer and Black Americans, pale, pearly-white skin became the symbol of the intelligent, wealthy, pure, and civilized (Tharps, 2017). In one interview with a Cham person–that is, an Indigenous and ethnic group along coastal Cambodia and central Vietnam from the original kingdom of Champa–Khmer was used as a derogatory term for dark Cham people, revealing dark skin tone associated with Khmer people and the negative implications behind having dark skin (Khanna, 2020). This stereotype contributes to dark-skinned Khmer Americans facing lower-paying jobs, lower socioeconomic status, and poor living conditions compared to the average Asian American population (Banh, 2023) and lighter-skinned Cambodian Americans (Jones, 2013), perpetuating a cycle of disadvantage and reinforcing the stereotype.

Existing literature suggests that ethnic identity impacts some aspects of the self, such as the ego or psychosocial functioning, entailing self-esteem, as belonging to an ethnic minority group is also associated with having resolved one’s commitments in occupational, educational, political, and religious domains (St. Louis and Liem, 2005; Phelps et al., 2001). Previous research has revealed a positive relationship between ethnic identity formation and psychosocial functioning for ethnic minority youth (Phinney, 1992). Ego identity formation is particularly critical in young adulthood, specifically during college, as identity determines the success or failure of adult development to an extent (Erikson, 1968). In turn, college students of color with well-developed ethnic identities have higher self-esteem and psychological well-being (St. Louis and Liem, 2005; Phelps et al., 2001). Ethnoracial identity has, therefore, been shown to be critical in influencing the educational experiences of students of color, which leads to the focus on my participants’ connection to Khmer culture as a key mediating factor. However, due to the negative stereotypes Cambodians face, many Cambodian students have made efforts to distance themselves from their ethnic group to perform better in school (Chhuon and Hudley, 2010), contrasting the literature deeming a positive relationship between ethnic identity and self-esteem and psychosocial functioning. Chhuon further discusses how, for Cambodian American girls, however, the norm was that they were studious and quiet in class, mirroring the model minority. This may be because women have a higher societal and cultural expectation to be obedient and do well in school than men. However, many Cambodian women living in the United States still view their community in a negative light, and my study thus seeks to observe whether or not ethnic identity influencing self-esteem would outweigh the negative stereotypes of Cambodians.

### 1.2 The influence of family

Family significantly influences self-esteem, especially in adolescence and early childhood. Family influence and support in students’ work are crucial for better academic satisfaction, career decision-making, and overall happiness in adolescence. Family guides them as major support even when peer groups and communities change (Fatma et al., 2023; Tahir et al., 2015). Family, especially the older female generations such as mothers and grandmothers, in particular, primarily aid in the process of racial socialization, where individuals view themselves as their racial group as well as gain behaviors, perceptions, values, and attitudes on that group (Kulish et al., 2019; Wilder and Cain, 2011). Consequently, ingrained feelings of colorism frequently appear from families giving messages to their children to protect them from the realities of racism, but in doing so, some families may also perpetuate colorism–a double-edged sword (Walker, 2014).

The role of family in colorism is deeply intertwined (Tharps, 2017), as it consists of the expectation of unconditional love, yet various treatments may be observed, especially if one has siblings of different skin complexions. Additionally, the family typically influences one’s development growth as they spend much of their time together growing up outside of school (Li and Guo, 2023). Landor et al. (2013) reported a higher quality of parenting, most likely due to the beauty standard favoring lighter skin. Women especially see themselves as a direct reflection of their mother, grandmother, or other female family member, as the maternal family members serve as the source of identity construction (Wilder and Cain, 2011). These studies demonstrate how ingrained colorism is for women and when another female figure is involved; thus, the significance of potential internalization of family support from female relatives.

Wilder and Cain's (2011) study found that some women credited the wide diversity of hues in their families for their oppositional foundations of colorism, whereas others described the color homogeneity within their families as causation for less color-conscious family experiences; thus, the focus on skin tone in the family as a theme in my study. Wilder and Cain (2011) also provided an example where, despite her parents’ adamant challenges to colorism, a participant’s subsequent exposure to extended family members, schools, and relationships overrode what her parents initially fought so hard to instill. This signals that social factors are interconnected; hence, my study posits the importance of analyzing the social factors, family and community, in tandem and its effect on self-esteem and views on one’s skin tone.

### 1.3 The influence of community

External and social factors, i.e., community, clearly demonstrate influencing self-esteem; for example, having an ethnic community or an online community consumed through social media may lead to the effect of self-racialization, identifying the dominant norms in appearance, and acceptance or rejection of the community membership influencing self-esteem (Steinsbekk et al., 2021; Umaña-Taylor et al., 2002; Phinney, 1991).

Harris (2013) article discusses how to feel accepted and validated within the Black community. The participants in a Predominantly White Institution (PWI) felt they needed to validate their community by taking classes, engaging in Black student groups on campus, and attending more campus-related events. Bhatt’s study with South Asian American emerging adults found general positive perceptions and experiences, and an increased sense of belonging with others of their ethnicity were the most common influences of skin tone on ethnic identity (Bhatt, 2023). These findings reveal a clear relationship between a sense of belonging and an ethnic community.

However, there is a lack of literature on the effects of specifically the complexion of the overall community beyond the ethnicity or race of the community on particularly self-esteem, much less focusing on colorism. Self-perception of one’s skin color is affected by the range of skin tones surrounding their social networks (Norwood, 2015). Mathews and Johnson’s, 2015 study discusses the difficulties of belonging for darker-skinned women due to their skin tone, even within the same ethnic community. With participants from Historically Black Colleges and Universities (HBCUs), darker-skinned women felt less valued in their friendships compared to lighter-skinned women. Notably, notable findings show that women who self-identified their skin tone as light provided the most egalitarian responses (Mathews and Johnson, 2015). Nambiar’s study on the experiences of colorism for South Asian women found that friendships and peers were a source of support for participants, largely when the times they felt great about their skin color were being with Black or Brown people who could also empathize with participants and not solely with other South Asians (Nambiar, 2023).

I attempt to address this gap in the effect of the complexion of the overall community on self-esteem among Khmer women in this study by accounting for how my participants compare their skin tone to those around them and not just other Cambodians.

## 2 Data and methods

This study consisted of qualitative interviews conducted from June to September 2020 involving a sample of 40 Khmer women. Due to the COVID-19 pandemic, travel was not possible. Accordingly, we conducted interviews over Zoom and Facebook Messenger to adjust to these new circumstances for the safety of all parties. Out of the convenience of video conferencing, we conducted interviews from a larger range of locations. While we expected participants to feel less connected or to face Zoom fatigue within the online setting, the majority of participants expressed eagerness to speak about their experiences, leading to interviews lasting longer than the initially suggested duration of 1 h.

Given the nuanced subject of colorism involving unique and complicated personal experiences, in-depth interviews allowed the required contextual information to further understand colorism’s factors and effects among Khmer women (Hays and Singh, 2012). Furthermore, both interviewers were Khmer American women, 20 years old at the time, and with medium skin tones. We both grew up low-income, but my co-interviewer grew up in Lynn, Massachusetts, with some Cambodian community, whereas I grew up primarily in the suburbs of Fairfield, Ohio, with a very small Cambodian community aside from my family. I have the darkest skin tone in my family, especially with sisters with pale skin from my mother’s part-Chinese ancestry, so my experiences of colorism were as early as my toddler years. I have grown up hearing and receiving comments about my dark skin from family, friends, Cambodians, and strangers alike. Colorism directly affected my self-esteem growing up, as well as my dating views. Visiting Cambodia multiple times and living there for 4 months, I have witnessed acts of colorism and the privileges that I also hold, being medium-toned and American. Despite the difference in higher education and immigrant generation from a handful of our participants, our positionality and ability to resonate with the participants’ stories made our participants feel more comfortable and open to freely sharing their thoughts and experiences.

### 2.1 Participants

We sampled and interviewed 40 Khmer women, where the only qualifications to participate in the study were to identify as ethnically Khmer or have Khmer ancestry to an extent, identify as a woman, and be at least 18 years old to consent. Of the 40 participants, 30 are Khmer American, in which they were either born in the United States or grew up primarily in the United States; nine are Cambodian, in which they were either (1) born in Cambodia and/or currently live there, or (2) grew up primarily, including adolescence years, in Cambodia, as opposed to 1.5-generation Cambodians, who were born in Cambodia or Thailand refugee camps and immigrated at a young age, identifying more as American; and one participant is Khmer French, having been adopted from Cambodia but raised in France with French citizenship. Participants’ demographics presented a wide range of variations in age, location, birthplace, relative complexion, and Cambodian community proximity, which can be referred to in Table 1. All names of participants are pseudonyms for their privacy.

**Table 1:** Participant demographic information.

Name (Anonymized)	Age	Current Location	Birthplace	Community Color^1^	Cambodian Proximity^2^
Angela	25–29	Ohio	New York	Darker	Not Many
Bailey	18–24	California	California	Lighter	Many
Bopha	25–29	Cambodia	Cambodia	Lighter	Many
Candy	18–24	Massachusetts	Massachusetts	Darker	Many
Caroline	18–24	Maryland	Cambodia	Lighter	Not Many
Chantha	25–29	California	California	Lighter	Many
Chea	45–49	Hawai’i	Cambodia	Lighter	Many
Chhaya	30–34	Cambodia	France	Lighter	Not Many
Dahlia	18–24	Massachusetts	Massachusetts	Darker	Many
Dara	35–39	Washington	Cambodia	Lighter	Not Many
Elise	18–24	France	Cambodia	Lighter	Not Many
Jamie	25–29	California	California	Lighter	Not Many
JJK	25–29	New Jersey	California	Darker	Many
Kalianne	30–34	California	Iowa	Darker	Many
Laura	18–24	Massachusetts	Massachusetts	Darker	Many
Lesly	25–29	Ohio	Ohio	Darker	Not Many
Lucy	25–29	California	California	Darker	Many
May	18–24	Texas	Texas	Lighter	Not Many
Michelle	25–29	California	California	Lighter	Many
Neary	45–49	Washington	Cambodia	Unclear	Many
Pam	30–34	California	California	Lighter	Many
Pich	45–49	Ohio	Cambodia	Darker	Many
Pisey	25–29	Cambodia	Cambodia	Lighter	Many
Rathana	45–49	Massachusetts	Cambodia	Lighter	Many
Reaksmey	25–29	California	California	Lighter	Not Many
Samnang	18–24	California	California	Darker	Not Many
Serena	18–24	California	California	Darker	Not Many
Socheat	25–29	Cambodia	Cambodia	Lighter	Many
Sokha	18–24	Washington	Washington	Lighter	Not Many
Sopheap	30–34	Wisconsin	Illinois	Unclear	Many
Soriya	35–39	Minnesota	Thailand	Lighter	Many
Sreyla	35–39	Pennsylvania	Ohio	Darker	Many
Sreyleak	18–24	Minnesota	Minnesota	Lighter	Not Many
Sreymom	50–54	Texas	Cambodia	Darker	Many
Tari	35–39	Massachusetts	Thailand	Darker	Not Many
Tevy	18–24	Cambodia	Cambodia	Lighter	Many
Theavy	18–24	Washington	Washington	Lighter	Not Many
Tida	50–54	Ohio	Cambodia	Darker	Many
Vanna	30–34	Cambodia	Cambodia	Lighter	Many
Wendy	25–29	California	California	Darker	Many
Total 40					

1 Categorized as either Lighter, where the community primarily growing up was either predominantly White and/or East Asian, or darker, where the community was either more diverse with predominantly Black and/or Brown.

2 Categorized as Many if they grew up with an extensive Cambodian community in their area, such as Long Beach, California; Lowell, Massachusetts; or simply surrounded themselves with many Cambodians aside from their family, or Not Many if vice versa.

Participants were initially recruited through snowball sampling and then recruited primarily through voluntary sampling as we promoted the study on our Khmer instructor’s Facebook page, which was then shared widely in a Cambodian-centered Facebook group. Only one participant in the sample was recruited in person with no personal connection at a Cambodian restaurant. The study’s budget, funded by the 2020 Chappell Lougee Scholarship, allowed all 40 participants to be compensated 30 USD for their time.

### 2.2 Procedure

Participants electronically signed an e-form sent via Zoom or Facebook Messenger chat to provide their consent to be interviewed and recorded. Additionally, we asked for verbal consent to be interviewed once the recording started, informed to avoid sharing anything unwanted, and with any identifying information redacted afterward. Interviews were initially conducted for storytelling purposes on the general premise of archiving Khmer women’s experiences with colorism. The interview duration averaged 1 h and a half and ranged between 45 min and 2 h, during which participants responded to 26 scripted questions, in addition to any follow-up or probing questions. Questions were reviewed by grant advisors and consisted of both closed questions to establish the participant’s positionality and primarily open questions to understand feelings or attitudes towards their skin color and facial features, colorist incidents or stories, skin tone concerning romantic relationships and children, occupations, and media representation—topics and concepts that have all surfaced or been researched in existing colorism literature as mentioned in this article’s previous section. With colorism as a social construct, participants self-reported their own skin tone description. I asked each participant how they describe their skin color relative to their family members. Because my study focuses on the effects of family and community on Khmer women predominantly living in the United States and Cambodia’s perception of colorism, my analysis refers to their skin tone relative to their family members unless specified otherwise. Interviews were primarily conducted in English, but all questions were translated into Khmer, and a Cambodian translator was present if needed, or the entire interview was conducted in Khmer, which consisted of six interviews. My co-interviewer and I can speak conversational Khmer. Often, English-speaking participants would say or be encouraged to say Khmer words or phrases to feel more comfortable and prevent phrases from getting lost in direct translation.

### 2.3 Ensuring quality

To maintain credibility and confirmability, I used triangulation through multiple analysts (Patton, 1999). While the study contained me as a coder and primary researcher, my advisor reviewed the codes at the beginning and end of phase one and the final codebook, alongside reviewing my reflection memos and reasonings behind each theme. In the first coding phase, both interviewers could identify patterns and numerous codes. To further reduce interpretive bias, after each interview, both interviewers would write reflection memos on interview summaries and highlights, patterns, and themes compared to prior interviews, as well as questions related to the study and implications to observe in future interviews. These all helped compare observations, identify the codes, and confirm and expand on existing colorism literature and personal experiences.

To maintain dependability and ethical validation, as another form of triangulation through multiple analysts, participants were aware of what topics and themes were going to be asked or discussed prior to the interview and were followed up with an email regarding the study completion, gratitude, and key findings (Patton, 1999). During this preparation, participants were informed that one of the study’s objectives was to observe the impact of colorism on their lives, wellbeing, and self-esteem, and interviewers elaborated on what this entails. Some participants followed up to chat more about the study personally, stating their connection and agreement to have more open dialogue and research on colorism in the Cambodian community. To use triangulation through multiple data sources (Patton, 1999), I sent participants their interview transcripts once we completed interviews or 10 months later as a courtesy for their reference or opportunity for reflection. Some participants would ask to redact certain information, usually personal or not applicable anymore, but none that affected study findings, and instead, they would reaffirm their stories.

To provide a further contextual understanding of the study, I analyzed the negative cases of my initial findings (Patton, 1999). Negative cases constituted participants undermining colorism, participants of lighter complexions not appreciating their lightness, and participants of darker complexions not minding their skin color when thought to be most susceptible. I examined negative cases by comparing interviews that deviated from the initial findings to consider why they did not fit into the pattern, and then I asked follow-up questions in future interviews about the negative cases. This led me to question the social contexts that may impact participants’ perceptions of colorism.

Therefore, I used critical race theory (CRT) as a framework and constructivist approach to interpret participants’ experiences through subjective interpretations that participants constructed through their experiences and not independently of the researcher (James and Busher, 2009). The CRT lens to colorism recognizes that with racism and other structural constants, colorism is socially constructed with hierarchies placed on skin tones within racial groups (Reece, 2019), further demonstrating skin tone as capital and a form of power and privilege (Hunter, 2002). Given my study explores the impact of social factors such as family and community as environmental forces, I use this approach informed by intersectionality (Collins, 2019) to account for contextual experiences where these social factors and structural systems in play coexist with and influence the participant’s individual experience they share.

### 2.4 Data analysis

All interviews were securely audio-recorded either on my cell phone’s microphone as a recorder or on Zoom, if applicable, with files stored in a USB hard drive. After completing all 40 interviews, I identified recurring patterns in which participants deeply reflected on the significant influence of family and community on their perceptions of colorism and self-esteem during their upbringing. Consequently, I developed a codebook to retroactively analyze the data and formulated research questions based on the existing interview transcripts. The data analysis using NVivo occurred once all interviews were conducted and not during the collection process due to time constraints prioritizing completing interviews.

The coding process consisted of two coding phases, with myself as a coder and my advisor reviewing the codes at the beginning and end of phase one and the final codebook. In the first phase of coding, both interviewers were able to identify patterns, and I thus created numerous codes from reviewing the interview transcripts three times. New codes were created by comparing other transcripts with a narrative from the transcripts as an example representing the code. Aiming for a deductive thematic analysis (Guest et al., 2012), I found that the most significant general themes consisted of (a) Family Support, (b) Skin Tone in the Family, (c) Community Color, and (d) Connection to Khmer Culture. I relied on existing literature on colorism outcomes to identify common themes such as self-esteem, skin tone preference in marriage, and skin tone preference in children to compare, as well as the explicit responses participants would give to specific questions. Using the codes from the first phase, the second phase of coding narrows in on specific yet common trends under each broader theme to identify any correlations or intersections, leading to a focus on four major social factors and their effect on Khmer women’s self-esteem. Then, the transcripts underwent a final round of review using the codes in the final codebook. I describe how I assess the four major themes in the following passages. My experiences with colorism in the Cambodian community also helped me identify trends and understand the data. The frequency of the sample size by theme, including self-esteem level, can be summarized in Table 2.

**Table 2:** Sample size by theme.

Number of participants				
Self-Esteem Level	Higher or Secure 14	Low Young but Secure Grown 13	Low or Insecure 13	Total 40
Family Support	Supportive 15	Unsupportive 13	Neither 12	Total 40
Skin Tone in Family	Lightest or Lighter 3	Middle or Same 16	Darkest or Darker 21	Total 40
Community Color	Lighter 22	Darker 26		Total 38
Connection to Khmer Culture	Feel Connected 25	Not Connected 13	Contextual/Complicated 2	Total 40

#### 2.4.1 Self-esteem

Self-esteem is measured in three categories: Higher or Secure, Low Young but Secure Grown, and Low or Insecure. Because self-esteem is dynamic and the level or feeling can change depending on the context, I determined which category based on the participant’s overall attitude when speaking about their experience during the interview. For example, if the participant would generally have much more negative feelings or associations, such as disliking their skin color or lacking a sense of belonging in general when talking about their skin tone or experiences, lack of worthiness or quality, or changes in mental health, I would code for Low or Insecure, and vice versa for Higher or Secure. If participants specifically discuss a shift in attitude, such as using phrases like “when I was younger…but now I feel…” then I would code for Low Young but Secure Grown.

#### 2.4.2 Family support

Family Support is measured in three categories: Supportive, Not Supportive, and Neither. Supportive would indicate the participant’s family, which includes both immediate and extended family, would provide them assurance, defense, or lessons on self-love for their skin color. Unsupportive would indicate the participant’s family would comment about their skin color, implying lighter is better, or dictate not going in the sun or offer lightening products. Neither would indicate the participant’s family was neither supportive nor unsupportive.

#### 2.4.3 Skin tone in the family

Skin tone in the family is measured in three categories: lightest or lighter, middle or same, or darkest or darker. Relative to the participant’s family, either immediate or whoever the participant considers family, the participant is coded into whichever category they deem themselves. Darkest in Family, meaning if they were either the darkest or among the darkest in their family; Lightest or Light in Family, meaning if they were either the lightest or among the lightest in their family; Middle or Same in Family, meaning if they were in the middle or had similar shades among their family and had been combined into one category given the greater similarity in experiences and results among participants.

#### 2.4.4 Community color

Community includes the overall complexion of the community members that the participants coded under Community Color, as well as if they grew up with an extensive Cambodian community in their area, such as Long Beach, California; Lowell, Massachusetts; or simply surrounded themselves with many Cambodians aside from their family coded under Cambodian Proximity. Community Color is categorized as either Lighter, where the community is predominantly White and/or East Asian, or Darker, where the community is more diverse with predominantly Black and/or Brown. Since some participants moved to different areas over time, their communities were coded based on where they primarily grew up, who they mostly spent time with or talked about, or where they considered their home and community. Two participants’ Community Colors were unclear, so they were removed from the analysis.

#### 2.4.5 Connection to Khmer culture

Participants were asked how connected they felt to Khmer culture to gauge their ethnic pride and were either categorized under Feel Connected or Not Connected. The question and response were very open-ended, and culture was defined; however, the participant personally defined it.

## 3 Findings

Every participant in the study has criticized or simply acknowledged how, generally, lighter skin, straighter hair, and a slimmer nose are heavily desired in the community, suggesting colorism within the Cambodian community, predominantly in the United States and Cambodia, persists as an issue. All but five participants received comments from other Cambodians and Cambodian Americans regarding their skin tone, either pointing out darker skin complexions or implying lighter skin is better. These actions are so common that when “…they call you ‘អើយអាខ្មៅ អើយអានេះអាក្រក់អា ខ្មៅ (hey blackie/darkie, hey this one’s so ugly, black)!’… it almost becomes a norm for Cambodian children or Cambodian kids, and we start to accept that,” as one of the Cambodian national participants, Neary, mentions, suggesting colorism as one of the few shared aspects of Khmer culture.

### 3.1 The impact of ethnic pride

I have heard [about when other Cambodians felt they were not good enough] from whitewashed Cambodians who were not [SIC] in touch with their culture. So I feel like they do not really know what they are talking about, especially about the culture that we share. Since they are not connected to it as much as I am, they view being Asian negatively. Since in America, you just want to be White, and you just want to be seen as an equal to them.

In her quote above, Laura, a Khmer American, suggests that one’s connection to Khmer culture affects one’s self-esteem and whether one views oneself negatively or positively. Similarly, Caroline, a Khmer American, shares, “that because I’m a darker skin tone, I kinda have engraved that like, ‘Oh, maybe I am more ethnically Khmer,’” implying that for some, darker skin is equated with being Khmer. Despite Laura’s suggestion that being surrounded by Cambodians or connected to Khmer culture would most contribute to self-esteem, there is no strong correlation between Khmer ethnic pride and self-esteem in this study, as displayed in Table 3.

**Table 3:** Khmer culture results.

Self-Esteem Level				
Culture connection	Higher or secure	Low young but secure grown	Low or insecure	Total
Feel Connected	10 (40.00%)	8 (32.00%)	7 (28.00%)	25
Not Connected	4 (30.77%)	4 (30.77%)	5 (38.46%)	13
Contextual/Complicated	0 (0.00%)	1 (50.00%)	1 (50.00%)	2
Total	14	13	13	40

#### 3.1.1 Complexity of Khmer culture

As culture does not have one concrete definition and because culture constantly changes, participants may have different ideas of what is included in Khmer culture, especially among different generations of Khmer people. For example, 10 participants discussed how these types of colorist comments are just a part of Khmer culture; it’s a norm. Sreymom, a Cambodian national, shares:

I was growing up when old people called me, “Oh aing khmao/*ឯងខ្មៅ* (you are black),” I did not feel it was racist, or I did not feel that I was offended by them calling me that. I just feel that “okay, old people, they just make fun of me; I let them make fun of me. They old I cannot do anything.” So, if you ask me how I feel about that, I can only tell you that. I can only tell you that’s how I felt when I was a kid. I never feel like, “Oh, that old lady is racist! Oh my god.” I never feel like that because it’s almost an acceptance because our culture does like that all the time to other people.

Of these 10 participants, the majority (*n* = 7) thus felt more disconnected and disliked their skin color as they engaged more with Khmer culture. Notably, two of the other participants love their skin tone but do not feel as connected to Khmer culture. Descriptors and nicknames such as khmao/*ខ្មៅ* (black), sraem/*ស្រអែម* (tan but in a beautiful manner), and sreymao/*ស្រីម៉ៅ* (black girl) were frequently used and received, oftentimes derogatorily, to where Elise, Khmer French, mentions that “There’s this whole culture of making fun.” Not only from blatant comments manifesting colorism throughout the interviews but also through actions and available opportunities, such as wearing long sleeves or having an umbrella on a sunny day, family members warning participants not to swim for too long in fear of tanning, the majority of participants (*n* = 33) not feeling represented in media and especially Cambodian media, participants (*n* = 16) trying whitening creams typically offered by mothers or Cambodian nationals, and feeling the need to work harder overall.

However, others talk about community, classical dance, holiday celebrations, and language as positive aspects of culture that allow them to feel closer to their Khmer identity, like Laura:

I really feel like I am a part of my culture because [my high school] has its own Khmer community, Khmer clubs, Khmer parties, and Khmer New Year’s. And then going to [university], there’s a Khmer culture there because of the clubs, and there are other Asian clubs I get to meet. We have a lot of Khmer things that go around, and you just see things, and you are just like, “Yes, this is where I belong.”

#### 3.1.2 Cambodian stigma

Several other participants discuss how a lack of a large Cambodian community contributes to a disconnect between them and their Khmer culture. Furthermore, over half of the participants (*n* = 21) discuss the stereotypes that come with being Cambodian, where Cambodians are the “ghetto Asians,” “the Black people of Asia,” “gang-infested,” “not being as achieving or as smart as other Asians,” “lazy or not hard-working,” “Cambodians are mean or Cambodians smell,” “lov[e] gambling and drinking,” “end up in jail post-high school,” and “low-income,” and this extends to Cambodian nationals and Khmer Sot/*ខ្មែរសុទ្ធ* (pure Khmer) as well, where “*ថាខ្មែរអត់ឆ្លាតដូចពួកចិនទេ* (Khmer people aren’t smart like Chinese people).” Three participants specifically mentioned how these stereotypes dissuaded them from connecting to the community and therefore disconnected them from the culture, like Bailey, a Khmer American, and her experience:

This is why I kind of separated myself from the Cambodian community. Because it was basically the stereotype that Cambodians were the ghetto Asians. We were the not-so-smart Asians. There were not a lot of Cambodians, particularly in my classes, AP classes, and accelerated classes. […] Definitely, now I realize what I did was stupid, but I definitely sort of tried to separate myself from my Cambodian peers because of that sort of stereotype that we are the ghetto, dumb Asians.

Kalianne, a Khmer American, and her quote below exemplifies how colorism is more than a beauty issue and the sentiment shared among the majority of participants about how life would be overall advantageous if they had lighter skin:

“Well, if you were white, if you were light-skinned, or if you were a lighter Khmer person, would you have that same confidence? You know, or would you navigate the world differently?” I would navigate it with much more confidence, I think. Because it would be easy for me to just get what I want, and I feel like I would not have to try as hard as I have to try now to fit in or, you know, get a job. <Laughs and sighs> Yeah, I just would feel more privileged.

In the next section, I analyze which combination of social factors contributes the most to lower self-esteem and higher or secure self-esteem, derived from the findings of each social factor theme. I find that family (*n* = 16) and community (*n* = 18) were the greatest contributors to how the participants viewed their skin tone, with media (*n* = 6) following behind, indeed signifying an influence on self-esteem and perceptions of colorism from the social factor themes. Thus, the following sections aim to understand how social factors, when together, can play a role in an individual’s experience with colorism and differ from another individual with a different combination of social factors.

### 3.2 Contributors to self-esteem

I specifically use semicolons to separate the conditions that more likely lead to having a low or insecure self-esteem. It should mean something like this: by possessing all of the following (1) Unsupportive family; (2) Darkest or Darker, or Lightest or Lighter skin tone in family; (3) and Lighter community and Many Cambodians. Thus, I pull the participants who possess all of the following themes to determine whether or not their self-esteem is low or insecure. I do not consider the theme when cross-tabbing the data because of the low or unclear significance of the Khmer culture connection shown above in Table 3.

Results of participants possessing all of the factors can be summarized in Table 4, which affirms the argument that having all three themes is more likely to lead to low or insecure self-esteem but demonstrates that these social factors have a greater influence on participants when they are young. Notably, no participant who had an unsupportive family was the lightest or lighter skinned in the family, possibly due to subconscious better treatment or having different expectations due to their lighter skin, as well as not having family point out their skin tone nearly as much compared to those darkest or darker.

**Table 4:** Contributors to self-esteem.

Self-Esteem Level				
Social factor combinations	Higher or secure	Low young but secure grown	Low or insecure	Total
Unsupportive Family, Darkest or Lightest Skin Tone in the Family, Lighter Community Color, and Many Cambodians	0 (0.00%)	3 (50.00%)	3 (50.00%)	6
Supportive Family, Middle or Same Skin Tone in Family, Darker Community Color, and Many Cambodians	4 (100.00%)	0 (0.00%)	0 (0.00%)	4
Total	4	3	3	10

In contrast, I observed that the following social factor theme conditions were more likely to lead to high or secure self-esteem: supportive family, middle or same skin tone in the family, darker community, *and* many Cambodians. Thus, I pull the participants who possess the following themes to determine whether or not their self-esteem is high or secure. All of their self-esteem levels were higher or secure, affirming the argument that having all three themes is more likely to lead to high or secure self-esteem.

#### 3.2.1 Social factor themes in tandem

**Figure 1:**
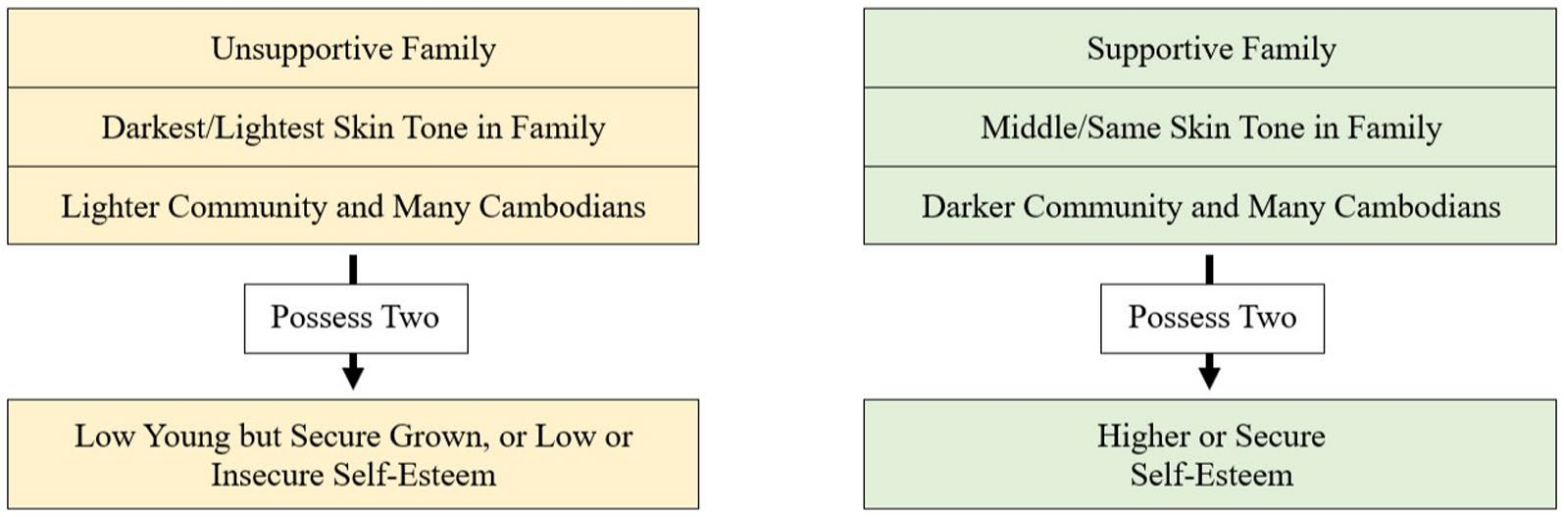
General trend for contributors to self-esteem.

Given the vast individual differences among the participants and their experiences with family, community, and overall values, the results of this study do not confidently determine whether and in what context one social factor theme consistently outweighs another theme. Therefore, I further examine how each theme contributes to self-esteem in tandem with one another rather than outweighing it, as each factor, aside from the connection to Khmer culture, has a substantial impact. However, they do not exist separately. Upon observing which combination of social factors contributes the most to lower self-esteem and higher or secure self-esteem, as seen in Table 4, I find that when combining at least two of any of the social factors theme conditions that most lead to lower self-esteem, the outcome of the individual’s self-esteem and perception of their skin tone will generally be lower. Similarly, when combining at least two of any of the social factor theme conditions that most lead to a higher or secure self-esteem, the outcome of the individual’s self-esteem and perception of their skin tone will generally be higher, as modeled in Figure 1. The following section elaborates further on how social factors in the context of family and community interact, affecting one’s self-esteem and experience with colorism.

### 3.3 The impact of family

[I]t’s kind of hard because it can be from your own parents, your own siblings, and that’s where I think it hurts more because it’s supposed to be people who are supposed to kind of uplift your confidence and boost your self-esteem, and not make you feel less than. Yeah, it’s definitely within the family.

Reaksmey, a Khmer American, and her quote above demonstrate how, because family is generally one of the closest relationships in their life, the support and treatment they receive will have a lasting impact on their self-esteem. Family dynamics are oftentimes internalized and compounded with visible differences between family members, such as skin tone, whether pointed out or not, frequently leading to a lack of belonging, which does not go unnoticed as Bailey reflects how “sometimes I feel [my sister] gets treated differently because of that, or I get treated differently because I’m just a few shades darker,” further elaborating:

I remember this comment that she made from that day. She said, “No matter what you do, you still look dirty.” And that’s something that really stuck with me throughout elementary school. […] I do not know [if] she would remember that, but that’s something that I will always remember. […] I think that was the first sort of instance where I just doubted why the color of my skin was the way it was. And, especially since my mom is kind of lighter-skinned.

Of the participants with a supportive family, the majority were secure overall or became secure at some point growing up, whereas those with an unsupportive family were insecure overall for a large part of their lives. The participants who were the lightest or lighter in their family, or darkest or darker, were overall insecure for a large part of their lives, whereas those who were in the middle or the same were overall secure or became secure at some point growing up. Findings can be summarized in Tables 5–7.

**Table 5:** Family support results.

Self-Esteem Level				
Family support	Higher or secure	Low young but secure grown	Low or insecure	Total
Supportive	9 (60.00%)	3 (20.00%)	3 (20.00%)	15
Unsupportive	1 (7.69%)	5 (38.46%)	7 (53.85%)	13
Neither	4 (33.33%)	5 (41.67%)	3 (25.00%)	12
Total	14	13	13	40

**Table 6:** Skin tone in family results.

Self-Esteem Level				
Skin tone in family	Higher or secure	Low young but secure grown	Low or insecure	Total
Lightest or Lighter	0 (0.00%)	1 (33.33%)	2 (66.67%)	3
Middle or Same	11 (68.75%)	3 (18.75%)	2 (12.50%)	16
Darkest or Darker	3 (14.29%)	9 (42.86%)	9 (42.86%)	21
Total	14	13	13	40

**Table 7:** Family support and skin tone in family results.

Self-Esteem Level				
Family support and skin tone in family	Higher or secure	Low young but secure grown	Low or insecure	Total
Supportive and Lightest or Lighter	0 (0.00%)	0 (0.00%)	0 (0.00%)	0
Supportive and Middle or Same	7 (87.50%)	1 (12.50%)	0 (0.00%)	8
Supportive and Darkest or Darker	2 (28.57%)	2 (28.57%)	3 (42.86%)	7
Unsupportive and Lightest or Lighter	0 (0.00%)	0 (0.00%)	2 (100.00%)	2
Unsupportive and Middle or Same	1 (33.33%)	1 (33.33%)	1 (33.33%)	3
Unsupportive and Darkest or Darker	0 (0.00%)	4 (50.00%)	4 (50.00%)	8
Neither Lightest nor Lighter	0 (0.00%)	1 (100.00%)	0 (0.00%)	1
Neither and Middle or Same	3 (60.00%)	1 (20.00%)	1 (20.00%)	5
Neither Darkest nor Darker	1 (16.67%)	3 (50.00%)	2 (33.33%)	6
Total	14	13	13	40

#### 3.3.1 Family support observed

Family support displays itself in multiple ways throughout the interviews, including defending or speaking back against colorist comments or actions towards the participant, being against whitening creams, participants feeling overall love from their family and therefore loving their features because their family gave them those features, and looking up to female figures in the family. The most critical determinants of family support were the type of comments about the participants’ skin tone and the amount of comparisons between and from family members. When participants are in the middle or have the same skin tone in the family, skin tone and comparisons between family members are less apparent and tend to be more understanding. May, a Khmer American, discusses how when she talks about colorism, her father “…understands what it’s like to be Brown because he is brown, but he’s not indigenous Khmer type brown, but he still understands, and he understands what they go through, and same with me.”

Negative or general comments from the family or self-awareness of appearing different in the family may increase feelings of insecurity. Soriya, a 1.5-generation Khmer American, shares:

I think with the teasing from the family, and how they put you down, it goes down all the way to the self-esteem because they’ll tell you, “Well, you have to go to college–Oh well, you are dark, you are so dark; you are not going to succeed in college, so why even waste your time to go to school. You’re not going to amount to anything anyway.” So then, we do not go to school, our self-esteem is so low, we do not believe in ourselves, so we just give up at the end, and then they wonder why there are no dark-skinned people in school or graduating high school.

#### 3.3.2 Comparing skin tones in the family

Oftentimes, comments by family members or self-comparisons between other family members played more of a role in how participants saw themselves, especially if compared to other female figures, either sisters or mothers. For example, Wendy, a Khmer American, says that she and her sister are the lightest, but she always thought her mom was beautiful, and her mom would always make comments and comparisons to her sister:

My sister, for example–we are half, so we look nothing alike–she takes after my mom; she has that smaller nose, she’s very light-skinned, she’s tall. She’s that ideal of beauty, and I’m seven years older than her, but people still think she’s the older sister. One confidence for sure, growing up as a young girl and you are still forming your ideas of what it means to be beautiful, it’s the little things…My mom likes to post on Facebook a lot, and she always says, “My beautiful daughter, blah, blah, blah.”

In Wendy’s case, it was clear that family was her biggest influence on how she perceived colorism, even despite being on the lighter side among her family. If participants were lighter, they were not the lightest among their family members, and typically, the lightest person was usually their mother or sister, never a male figure. Seventeen participants spoke about having a lighter parent, of which all but one were the mothers.

#### 3.3.3 Family support and skin tone in the family

Despite an obvious difference in complexion, some participants were able to still appreciate their skin color, differing from the overall trend of being darkest and lightest in the family, leading to lower self-esteem due to positive reinforcement from that parent, demonstrating the importance of family support as Lucy, Khmer American, points out:

With my parents, I am a little bit tanner than my mom is, but she does not really discriminate against me because of that. If anything, she tells me that my skin color is beautiful and that others should be envious because while others are going to try to get tan and stuff, I have a natural tan.

JJK, a Khmer American who has a lighter parent and lives in a lighter-skinned community, further affirms the notion that a supportive family is essential as she feels “…very lucky that [she] had a pretty nurturing environment, so [she] never hated [her] skin or ever wanted it to be a different color.” However, when there was a lack of support from family, many participants instead would look towards their community, including non-family members, as a form of support.

### 3.4 The impact of community

My support system was not my family. My support system was actually other Black people. That’s the only way that I survived in school. It was not other Cambodian people or my family. I had to look outside of my race, and I had to look outside my skin color and everything to get that support. I did not graduate from high school due to mental illness. Mental illness that’s like generational trauma during the Pol Pot regime and everything like that. So, I had to deal with that, plus I had to deal with colorism, and so I had to deal with the family trauma of all that. So, layering all that put together, going through therapy, and having a family that has no type of understanding of any of that.

Similar to Soriya, a Khmer American, in her quote above, many participants spoke of the impact of family as very internal, especially with the intergenerational trauma that accompanies second-generation Cambodian immigrants, while community is external and oftentimes a substitute or alternative for family support. However, successful substitutes and higher self-esteem tend to occur when the community consists of people of darker skin tones. Lighter-skinned communities, on the other hand, have rather more complex outcomes.

The majority of those with a lighter-skinned community were overall insecure for a large part of their lives, whereas the majority of those with a darker-skinned community were overall secure or became secure growing up, as seen in Table 8. Given that Khmer people are typically darker in skin tone compared to White and East Asians, many participants did not feel like they fit in with lighter-skinned communities, hence why participants like Soriya were able to find support in their darker-skinned communities. Jamie, a Khmer American, was one of the only Cambodians in her high school, but not the only Asian. She recalls feeling excluded among the Hmong students at her school:

**Table 8:** Community color results.

Self-Esteem Level				
Community color	Higher or secure	Low young but secure grown	Low or insecure	Total
Lighter	3 (13.64%)	9 (40.91%)	10 (45.45%)	22
Darker	10 (62.50%)	4 (25.00%)	2 (12.50%)	16
Total	13	13	12	38

Outside of that, too, growing up because the other Asians at my school were Hmong, and when Americans think of Asians, they think of small, pale-skinned Asians, slanted eyes, really silky thin hair, and straight hair, and I did not look like that. That’s how the Hmong kids looked like at my school, and I already wasn’t accepted by them because I was not Hmong, and then I was also just a different-looking Asian. I did not feel like I fit in with other Asians, and then, within mainstream media, I did not look like the stereotypical Asian. It’s kind of a mind fog like [I ask myself], “Are you Asian at this point?” I do not hang out with them, all my friends aren’t Asian, and I do not really look like what people expect me to look like as an Asian person, so it messes with you.

There is no direct connection between having a Cambodian community and the participant’s self-esteem. This may be due to the fact that while one can feel a sense of belonging while surrounded by Cambodians, it can act as a double-edged sword as many Cambodians reinforce the notion that lighter is beautiful as Tari, a 1.5-generation Khmer American, says:

When you are hanging out with friends, they are like, “Oh, she’s ខ្មៅជាងគេ (the darkest/blackest),” or like, “Oh, she’s the darkest one.” I mean, I catch myself saying that when somebody is darker than me, I’m like, “Oh yeah, she’s darker than me,” or you know, you use skin color to describe someone. But I was always taught that lighter is just more beautiful.

#### 3.4.1 Being the darkest in the room with or without Cambodians

Participants also discussed how their skin tone felt more salient when surrounded by lighter-skinned people, regardless of whether they were Cambodian or not. May notes that “Cambodian people always comment on how [she’s] dark” but “never had a person darker than [her] point out [her] skin color.” Therefore, I also analyze the effects on self-esteem when combining Community Color and Cambodian Proximity into four categories: Lighter and Not Many Cambodians, Lighter and Many Cambodians, Darker and Not Many Cambodians, and Darker and Many Cambodians. The majority of those with Lighter and Not Many Cambodians were overall insecure for a large part of their lives. In contrast, the majority of those with Darker and Many were overall secure for a large part of their lives (Table 9). Findings can be summarized in Table 10.

**Table 9:** Cambodian proximity results.

Self-Esteem Level				
Cambodian proximity	Higher or secure	Low young but secure grown	Low or insecure	Total
Not Many Cambodians	6 (40.00%)	4 (26.67%)	5 (33.33%)	15
Many Cambodians	8 (32.00%)	9 (36.00%)	8 (32.00%)	25
Total	14	13	13	40

**Table 10:** Community color and Cambodian proximity results.

Self-Esteem Level				
Community color and Cambodian	Higher or secure	Low young but secure grown	Low or insecure	Total
Lighter and Not Many Cambodians	3 (30.00%)	2 (20.00%)	5 (50.00%)	10
Lighter and Many Cambodians	0 (0.00%)	7 (58.33%)	5 (41.67%)	12
Darker and Not Many Cambodians	3 (60.00%)	2 (40.00%)	0 (0.00%)	5
Darker and Many Cambodians	7 (63.64%)	2 (18.18%)	2 (18.18%)	11
Total	13	13	12	38

Having many Cambodians in the community but having them lighter than the participants in skin tone made several participants insecure. They see that this should be their community, and so they should feel like they belong, but because they do not look alike or experience the same issues of colorism because of their different skin color, participants are even more aware of their skin color and feel insecure in comparison to participants who have a large Cambodian community who are also just as dark but are overall more Higher or Secure. Dara, 1.5 generation Khmer American, for example, shares:

I was insecure about my skin color, both when I was growing up, just because I was surrounded by White people, and then having the Khmer people telling me that my skin is dark.

They mainly felt self-conscious about their skin color or no sense of belonging whenever they noticed they were the only brown person in the room or when they were in Asian spaces but noticed that a vast majority are ethnically East Asian, Cambodian or not, and thus a lighter Asian. Rather than what is said that hurts the interviewees, it’s what is not said that hurts more. Pam, a Khmer American, recounted moments in her Cambodian student organization at her university:

I’m like the darkest, so I really stand out, and I have noticed that any pictures someone posts or something you do like differently, they’ll be noticed for it like, “Oh yeah, they are really beautiful,” and I do not think I’ve ever been told I was beautiful. So, most of the time, I do not like my skin color. I think I actually thought about [how] people use those papaya soap and to kind of just lighten up their skin.

Pam’s comment demonstrated that even having other Cambodians as friends or around you will still have the interviewees question their value and worth if they constantly notice that the Cambodians around them are lighter and receiving more compliments or opportunities. Candy, a Khmer American, had a secure and high self-esteem and sense of belonging, yet still commented that the only time she felt negative feelings surrounding colorism and her skin tone was when she was with other Cambodians because they had lighter skin:

[It was] just whenever I was in an environment that did not have any dark Cambodians. Before my church, I felt like they were all Cambodians, but they were all super light. Whenever they would talk about skin color, they pointed out me, my brothers, and my dad. […] I did not feel alone on it, so it did not take a toll on how I felt about it much, but that would really be it.

The sense of belonging difference asks what an ethnic community looks like or feels like with people of different skin tones and experiences. Especially when in lighter-skinned communities, comparisons that were between family members also opened up to peers where participants discussed an example of peers holding up their arms to compare skin tones, in contrast to a participant with a supportive family and able to see representation within their darker-skinned community, like Lesly, Khmer American:

I love that my family has so many different colors, so it’s not just everybody was the same color. Even living in the community that I was growing up in has always been somewhat diverse. I did not have to worry about it. I know I have my friends out there in the community, and then I would have my family, too. I can always have their support.

Observing participants who grew up in a lighter-skinned community, despite having a supportive family or being the middle or same skin tone in the family, and still having lower self-esteem additionally illustrates the impact of having at least two Social Factor Theme conditions to better understand the outcome of the self-esteem, referring back to Figure 1.

#### 3.4.2 National context

Notably, diversity may look different depending on the national context in which a diverse set of racial groups in a community may be more prevalent in the United States, for example, than in Cambodia, where diversity is more seen in skin complexion and ethnic groups rather than race.

I saw it more in Cambodia than in the US because…well, for my mom, it would be to stay out of the sun and wear more clothes so that your skin does not get dark. And then maybe she had a couple of lotions, but she never tried any, never pushed any skin-whitening products on me…But then, when I was in Cambodia, I remembered my cousin. One of them showed me a bottle, and she was like, what bottle is this? The person who sold it to me—so the text is all in English—the person who sold it to me said that this was it will make your skin lighter. I read it, and I think it was just regular sunblock. […] That same cousin wanted to […] lighten my skin through a milk bath. And I was like, “No, that’s okay.” But then, being in Cambodia or watching Asian shows, you would see commercials for skin-lightening products.

Similar to Dara’s experience above, 27 participants discussed comparisons between experiences in the United States versus in Cambodia, as several participants have visited Cambodia in the past, and eight participants currently live or grew up in Cambodia. Due to the differences in race and identity politics between the United States and Cambodia, given the idea that the United States is more heterogeneous in terms of race while Cambodia is more homogenous, the influence of community can look and mean very different. However, the interviews reveal that Cambodia, while homogenous in terms of race compared to an American context, consists of varying ethnicities and, therefore, skin tones, affecting how participants viewed their own skin tone accompanied by the more tangible notion that lighter skin is superior given the more extensive application and push to use whitening creams, and blatant and direct comments on skin tone in Cambodia, as over half of the participants remark. In Cambodia, Socheat, a Cambodian national, said the way they talk about people, they “only have two standards: saw/ស (white) and khmao/ខ្មៅ (black).” Comments and being compared by people anywhere in Cambodia were the largest reasons behind frustration and insecurity. The social pressure to appear white even outweighs family support in Cambodia as Tevy, a Cambodian national, said that despite her family loving her:

My mom would try to get me to use it and buy me that stuff, but by the time I was 12, I moved to live with my sister, and my sister would not ever allow me to use any of those, so I did not really use it up until I was in university. And then, I was living with many girls and felt pressured, so I’d just use some moisturizer, but I only used moisturizer.

The findings demonstrate how a national context affects a group, primarily focusing on the Community social factor. American participants also confirm Cambodia’s values of lightness where Sopheap, a Khmer American, talks about how in Cambodia, with her cousins, she was not expected to cover up as much because of her American nationality:

Even though it’s 104 degrees, they are still wearing long sleeves. I was wearing a tank top, and then our cousin tried to wear a sleeveless one, and her husband and everybody else started yelling at her. “What do you think you are doing?” She’s like, “It’s hot,” and they are like, “Go put on clothes, you are naked.” I’m like, “Oh, am I naked?” but they are like, “Oh, you are fine,” I guess ‘cause I’m American, I’m not really Cambodian then, so she had to run back and put on.

Elise, who grew up in France but moved and traveled frequently around Asia and America, stated, “In Asia, you feel the color of your skin 10 times more than in the US or, for example, France.” Despite Cambodia’s population being often seen as “homogeneous” in comparison to the United States, racism and colorism are still widespread issues, especially for women. Socheat even brought up a testimony where she was almost not hired for her job:

[T]here were two interviewers. One of them, who’s my current boss, told me they chose me because they liked me but the interviewer did not want me because I was too dark and they told my boss “You can choose her if you want, but I do not want her.” My boss works under the other interview. We (the other candidate and I) were evaluated based on our experience and our appearance. They wanted someone who was good-looking.

In the United States, for some participants, the racial heterogeneity of vast shades could help love one’s skin tone but could alternatively lead to feeling fetishized by White people for their darker skin tones.

The capital city of Cambodia, Phnom Penh, has a significant proportion of the Chinese and Chinese-mixed Cambodian population, and therefore lighter-skinned Cambodians. Cambodian participants would frequently mention how they were often the darkest ones in the room once moving to Phnom Penh. This factor is crucial, as nearly all talked about repeatedly receiving comments comparing and pointing out their dark skin or that it’s ugly to other Cambodians, furthering the strong influence of having a lighter-skinned community and many Cambodians. In the case of Socheat and Tevy, whose self-esteem grew over time, they talked about how this change happened after being exposed to more diverse people and international ideas of beauty. Socheat, for example:

I used to have a Black professor. As far as I’m concerned, they were from America, and they taught at a university here. They once asked me, “Why do Cambodians like to cover themselves so much? They wear socks and gloves out under the blazing sun. In America, people do not like that (fair skin). They like dark skin. They like tanned skin. Why do Cambodians like to hide (their skin) so bad?” That’s what they said. It made me think, why do we like to torture ourselves like this? Whether you bleach your skin or use whitening lotion, you will end up destroying your skin. Why do not we love our own skin (color)? When [the professor] said that, I started to think, why do we let others’ words influence us? Why do not we use [SIC] natural ways, like sunscreen, to protect our skin? This should be enough. Why do we have to torture ourselves to the point where we cannot sleep?

I infer that this could be due to the idea that these five women do not feel like they have a community. When you are in Cambodia, there are going to be many Cambodians, but instead of having a close community where people are supposed to look like you, relate, and support each other, they see people who are much lighter, who do not look like them and instead start comparing one another as well as making rude or blatant comments about their dark skin. This false sense of community and just feeling othered in your own community can feel isolating, and people lack a sense of belonging. Bailey talked about how, because of the norm of Cambodian Americans being ethnically mixed, being darker made her stand out and “feel ostracized,” hence the importance of representation–seeing people who look like you but also who have high self-esteem and love their skin tone. Socheat also talked about the difference in media from Cambodia to American media:

Another thing is the media in Cambodia. Sometimes, I watch foreigners on Google. I do not know what ethnicity they are, but I think they look really pretty with their dark skin. I look beautiful in their clothes and makeup. I’m just wondering why Cambodian media does not like dark skin. Like Rihanna, Alicia Keys, and Beyoncé, they have dark skin, do not they? But they are popular. They’re really pretty, right?

Sreyleak, a Khmer American, also informs how constantly seeing lighter-skinned Asians and people informs her view on her skin color in her anecdote:

But when I was in middle school, back when I really liked K-pop—I think the big theme here is how K-pop and Korean media, or the absence of Asian American representation in American media, pushed me into finding media elsewhere, and that media was Korean media—I used to put so much effort into trying to get my skin to be lighter.

While colorism is seen as more overt in Cambodia than in America on a surface level, especially with the frequency of people suggesting to darker-skinned women that lightening creams should be used and blatant comments disliking darker skin, colorism in America is also complex and appears in similar ways, but in addition to navigating the Cambodian American cultural identity and community.

### 3.5 Breaking the cycle

I make it a point to this day to never say anything to my daughters about anything with their looks or self-esteem, nothing about their skin color, because it should not matter. Because I know how it affected me as a child.

As family support is malleable, 16 participants in the study, like Soriya in her quote above, shared, unprompted, their efforts to prevent similar feelings of disliking their skin tone and experiencing colorism to the future generations such as their children or other Cambodian or Cambodian diaspora youth. Efforts include not making statements or comments on skin tone, including microaggressions, and instead having positive reinforcement regarding skin tone; raising or emphasizing Cambodian pride and being proud of their skin color; surrounding themselves with people who love their skin tone; teaching their children to be actively anti-racist and anti-colorist or to treat others well regardless of skin tone; and providing education and awareness on the systemic issues and history behind colorism and racism, as well as Cambodian history that shaped this beauty ideal rooted in classism in Asia, and exacerbated by colonialism and influenced by Eurocentric beauty ideals. Participants suggest that efforts to break the cycle of colorist norms for the future generation require more action and less complacency, as Dahlia, a Khmer American, urges:

What I would want to change is just going forward, how we interact with each other, how we treat each other, and how we destroy the notions in our minds that we have grown so comfortable with. Just do better going forward instead of trying to change what’s previously been implemented in the past. I feel like that’s the duty of every Khmer person, whether you are affected by colorism or not. You need to do your part to make sure that we do not repeat past mistakes. It’s people’s lives that are on the line at some point, like Khmer Angkor; they are just not as present as they used to be due to the treatment that they have received, and it’s just like they are dying out. That’s happened because we were so neglectful as a country–not as a country, as a whole, as a community as a whole. But yeah, it’s just actively being anti-racist, anti-colorisist, and not just standing by and hoping that somebody else will do the work because I feel like that’s a big problem.

Overall, the majority of participants strongly encouraged more dialogue surrounding colorism, especially to bring awareness. Once Michelle, a Khmer American, learned the word colorism, she realized “That there was a word for it and that there were other people who went through the same thing and that we could talk about it.” Chantha, also Khmer American, only felt able to “confess” her experiences of colorism, anti-Blackness, and using whitening products once she “realize[d] others have experienced it too.”

As some participants seek to make efforts for future generations to encourage a more positive view of their skin tone, some participants in the past actively sought to distance themselves from their Khmer identity to avoid negative attributes of the culture and identity that have, or they feel will hinder their growth and success. However, many Cambodians within and outside the study have expressed a desire to rekindle or preserve Khmer culture. A large portion of participants expressed regret at not learning the language growing up or having never gone to Cambodia, with the thought that because the Cambodian population is already so small in diasporic countries, there is a feeling “to keep the culture alive and keep it going,” as Jamie puts it. Additionally, because so many second-generation Cambodians initially assimilated into the dominant culture, losing their cultural identity, participants like Caroline shared their concerns:

I wish I were more connected. I’m kind of like on my part where I want to be more connected and celebrate culture more, but I do not know how to, and I do not want to be disrespectful to the culture and celebrate in the wrong way. But, like, I tried to celebrate the Khmer New Year, and I tried to do all that, but there’s definitely a disconnect because I did not grow up with culture, and I just do not know anything about it. And I’m trying to learn, but it’s also really hard to learn, like all these traditions, without witnessing it first.

Furthermore, culture continuously changes, even reflecting on the current differences between Cambodian and Cambodian American cultures. This raises the question of how and what to preserve in the culture, especially taking colorism into account as a part of Khmer culture by some Cambodians and other negative aspects.

## 4 Discussion

### 4.1 Colorism within the Cambodian community

This study reveals that colorism perseveres in various ways in the Cambodian community, predominantly in the United States and Cambodia. Through examining the pervasive presence of colorism within this community, the complex interplay between social factors becomes apparent and collectively affects participants’ self-esteem and perception of their skin tone, highlighting the intricate nature of the interactions between family, community, and cultural influences.

Due to various definitions of culture and the stigma of being Cambodian, connection is also subjective, emphasizing the need to examine multiple social factors. This continues the exploration of how an individual’s sense of ethnic identity varies greatly when the community possesses diverse skin tones and experiences. The complexities of a cultural connection thus further reveal the importance of considering family support, skin tone within the family, and the complexion of community alongside cultural connection.

Findings suggest family support holds significance, especially during the younger or critical developing portion of participants’ lives, as having a supportive family during this time will lead to secure self-esteem in the long term; having an unsupportive family will lead to low or insecure self-esteem more long term; and having neither demonstrates some kind of malleability in self-esteem and growth later on in their lives, possibly due to independence developed from not having family play a large role in colorism. The majority (8 out of 12) of participants with neither a supportive nor unsupportive family fell into the low or insecure and low young but secure grown categories, thus demonstrating the importance of putting in an effort to be supportive. There is a difference between being supportive and being not disrespectful or non-supportive, as the lack of family support generally leads to lower overall self-esteem. Of those who receive little to no family support or have a strained relationship with their family due to numerous negative comments on darker skin tones or comparisons among family skin tones, many have low self-esteem.

In addition to the evidenced structural effect of colorism in the Cambodian community, this study reveals the impact of social factors as reference groups that contribute to and/or exacerbate Khmer women’s perceptions of colorism. Given that life experiences and emotions are a result of a combination of various factors, including internal and external, it is critical to acknowledge that while a social factor can significantly contribute to how an individual feels about their skin color, it is highly unlikely one single social factor completely attributes to that feeling, especially as self-esteem is contextual. Therefore, to determine which factors contribute to self-esteem and to what extent, I analyzed the data on how each of the four social factors interact with one another: (a) Family Support, (b) Skin Tone in the Family, (c) Community Color, and (d) Connection to Khmer Culture, and their impact on self-esteem.

Through analyzing four Social Factors Themes–Family Support, Skin Tone in the Family, Community Color, and Connection to Khmer Culture–together and their impact on self-esteem, the combination of two or more themes that lead to a Lower Self-Esteem (Unsupportive Family, Darker/Darkest or Lighter/Lightest Skin Tone in the Family, and Lighter Community Color and Many Cambodians altogether) generally resulted in lower self-esteem and a more negative perception of one’s skin tone. Similarly, the combination of two or more themes that lead to a Higher Self-Esteem (Supportive Family, Middle or Same Skin Tone in the Family, and Darker Community Color and Many Cambodians altogether) generally resulted in higher self-esteem and a more positive perception of one’s skin tone. As seen through the Connection to Khmer Culture theme, the individualized and contextual interpretation of culture and one’s connection to it demonstrates how context within the family and community can either shape a negative or positive experience for both one’s ethnic identity and skin tone. As such, the combination of two or more themes contributing to self-esteem suggests that these social factors work in tandem rather than one theme consistently outweighing another and consequently demonstrates the complexity of interactions between family and community.

This study further reveals the structural hierarchy of lighter skin at the top and darker skin at the bottom, hence why Chinese immigrants and Chinese Cambodians are typically viewed as and hold a higher social standing than Khmer people. When speaking of the most marginalized populations in Cambodia, participants, both Cambodian nationals and not, would generally refer to Cambodians working in the countryside, typically as farmers or poor, referring to Khmer Sot/ខ្មែរសុទ្ធ (pure Khmer) or the darkest Cambodians on the spectrum.

Moreover, almost half of the participants (*n* = 19), all living outside of Cambodia, reported being frequently misracialized or misethnicized by others, often as Black, Hispanic, Filipino, or of mixed race. This highlights not only the inability of societies, particularly outside Cambodia, to identify Khmer individuals or recognize their physical attributes accurately but also underscores the existence of a racial and social hierarchy. This hierarchy, well-documented in the literature (Yip et al., 2019; Willmott, 1981), places Southeast Asians at the bottom and East Asians at the top within the Asian racial group, aligning with American society’s stereotypical image of an Asian person, which often resembles East Asian phenotypes.

### 4.2 Comparisons across previous literature

Despite the notion that colorism is more blatant and overt in Cambodia than in America or other diaspora countries (Khanna, 2020), findings suggest experiences of colorism remain quite similar and almost universal within the Cambodian community. While colorism may feel more overt in Cambodia due to the larger presence and pressure of using whitening creams and direct comments on skin color, participants in the diaspora countries also speak of their experiences with whitening creams and, to the same extent, with direct comments on their skin color from family members or the greater Cambodian community. The persistence of colorism in these countries may manifest in various ways, though still stemming from the mainland of Cambodia, and thus frequently appear in similar actions, such as the acknowledgment of or desire for lighter skin, straighter hair, and a slimmer nose for better social and economic opportunities; receiving comments or derogatory nicknames based on their skin tone; demands from others, such as wearing long sleeves or not being out in the sun for too long; not feeling represented in Cambodian media aside from low roles such as comedies, as farmers, or as servants; and receiving offers and using whitening creams.

Colorism in Cambodia may also feel more overt due to the larger presence of diverse racial groups in diaspora countries like the United States acting as a buffer and seemingly more tolerant or accepting of various skin complexions, though more so by individuals outside of the Cambodian community. Notably, this does not dismiss the fact that colorism within the Cambodian diaspora community persists in similar ways. The conversation regarding comparisons of a national context implies the significance of communities in play, as well as the ongoing colorism throughout all generations, implying how colorism is passed down in the family. This study highlights the persistence of colorism within the Cambodian community, specifically at the expense of indigenous or ethnically Khmer women. It emphasizes the importance of family and community dynamics in shaping the self-esteem and experiences with colorism of individuals not only within the Cambodian community but also in communities of color as a whole.

In America, tanning shifted from a symbol of labor and work into leisure and wealth in the 20th century (Martin et al., 2009), yet the study’s findings of consistent colorism within the Cambodian community demonstrate the nuances of colorism as well as implications of who benefits from tanning or darker skin. As the Pan-Asian beauty ideal in Asia and racial ambiguity in the United States become increasingly popular (Strmic-Pawl et al., 2021; Yip et al., 2019), studying Khmer American communities and other Southeast Asian American communities becomes relatively important as they live in in-between states. While colorism in East Asia is more deeply rooted in classism (Rondilla and Spickard, 2007), Southeast Asians like Cambodians—as seen in the findings—are more negatively racialized, even sharing a similarity in skin color hierarchy in the Philippines (Rondilla, 2012). The average Khmer person’s skin is typically a shade in between White people and Black people (Willmott, 1981), bringing the question of where their privileges and discrimination lie and how they navigate their identity in countries outside of Cambodia. The parallels between the Black community with stereotypes as being troublemakers, gang-associated, unintelligent, aggressive, or violent (Monk, 2014; Maddox and Gray, 2002) and the Cambodian community, primarily given due to their darker skin, broader facial features, and hair texture, signify how understanding colorism in one particular community may also contribute to understanding colorism as a whole and in efforts towards solidarity despite differences in history, including colonial and imperial patterns.

### 4.3 Implications for future generations

The conditions of the social factors that lead to either a lower or higher self-esteem exhibit the magnitude of supportive social networks and cultural pride, which are tied to a sense of belonging. Whenever the reference group is lighter in skin tone, regardless of the skin tone relative to society, self-esteem and sense of belonging decrease; this is especially true when faced with a larger Cambodian community, as participants’ expectation to feel a sense of belonging is higher, thus causing deeper infliction when faced with differences. Possessing a supportive family or community, however, would mitigate these feelings to a certain extent, though not entirely, especially when in environments with typically stable factors such as being the darkest in the family, having a lighter community, or simply being a darker-skinned individual in a society that values lighter skin. By finding support within the family and community, individuals may be able to resist and navigate the negative effects of these structural hierarchies, especially with the compounding impact of globalization and the legacies of colonialism and white supremacy, fostering a more positive self-image of their skin tone and themselves as a person.

Understanding the social factors prevents history from repeating in the community and continues the conversation about colorism and how to move forward. The literature discusses how common African American families introduce and reinforce messages that depict racialization yet can lead to a greater understanding of racial pride (Walker, 2014), in contrast to participants’ experiences where family members generally provide negative comments on their skin tone; thus, the desire or will to provide positive reinforcement for the future generation highlights the actions against colorism moving forward.

Moreover, the study confirms that colorism is a complex and nuanced structure that needs to be further researched to be understood. Not only should Cambodian communities take note of the study’s results in addressing colorism within the community, but other communities that face colorism should do so as well, given the similarities between other studies, to move toward racial justice. Despite documented colorism, which has existed for at least thousands of years (Dixon and Telles, 2017), the issue has only been widely discussed relatively recently. Even some participants had never heard of the term colorism before the study or simply possessed no words to describe their experiences. After gaining knowledge of the word colorism and others’ similar experiences, participants were inspired to engage in conversations with one another, highlighting the importance of awareness and dialogue in racial and ethnic justice efforts.

Furthermore, participants felt more inclined to share their experiences of colorism, anti-Blackness, and using whitening products, signifying the internal shame that accompanies colorism and latches onto darker-skinned individuals in attempts to position themselves higher in this pre-existing hierarchy. The negative comparisons between dark-skinned participants and Black people and actions and mindsets to withdraw from Blackness in this study demonstrate the inherent anti-Blackness driving colorism within the Khmer community. Providing a better understanding of the dynamics and consequences of colorism aids in initiating conversations and implementing anti-discrimination measures and educational programs necessary to challenge and address this issue. It is imperative to promote positive body image, media representation, and self-acceptance and foster supportive environments that value diversity and reject discriminatory gender and beauty standards for the sake of mental health, self-esteem, and racial justice.

### 4.4 Limitations

Interviews were initially conducted for storytelling purposes on the general premise of archiving Khmer women’s experiences with colorism, with current research questions formed and analysis conducted retroactively; thus, this study contains limitations where findings could have been formed more precisely. Conducting follow-up interviews in future research would aid in ensuring quality. As interviews were conducted during the 2020 Covid-19 pandemic lockdowns, the environment affected mental health and well-being through isolation, loneliness, and other material losses (Hwang et al., 2020), potentially heightening participants’ emotions and attitudes towards their experiences with colorism and self-esteem. Although this qualitative study required each participant’s individualized experience to benefit and further contextualize the study’s objectives, the subjective nature of self-esteem made it difficult to compare data among participants’ varying levels and thus potentially alter findings.

I acknowledge that skin tone is a nominal category that varies based on context and that complexion changes over time, representing another limitation of the study. When participants share their experiences, their descriptions of skin tone and complexion are contextual and ultimately self-reported, which may influence how they perceive their skin tone in relation to other Cambodians. This underscores the social construct of colorism while acknowledging a shared understanding of what is considered a dark complexion or “too dark,” given the racialization of Khmer people and the perception of dark-skinned Khmer individuals from rural areas as occupying the lowest position in Cambodia’s social hierarchy.

Additionally, due to the sensitivity of colorism and the historical trauma of censorship within the Cambodian community—rooted in the legacy of the Khmer Rouge—responses may have been curated or biased, whether to save face or mitigate potential retaliation. Even if this were the case, the responses still demonstrate what is considered politically correct or “normative” in the Cambodian context, revealing the pervasive impact of colorism and its intricate relationship with social context and reference groups.

The use of snowball sampling in this study introduced a high susceptibility to bias, as participants were recruited based on their interests, leading to less control over the sample composition. For example, we received many participants living in California and not as many participants over the age of 60 years. Additionally, there may have been a bias toward American interviews, as these tended to last longer compared to those with non-American participants. One French national participant was also considered an outlier. Given the sample size, the perspectives of Cambodian nationals and non-Americans, while consistent and substantial within the findings are limited. Further investigation focusing on a non-American Khmer sample and targeting intentional age groups with a larger sample size would strengthen and further the narrative and literature, especially on the impact of community and national background. Given the complexity of Khmer culture found in this study and the close associations between ethnic pride and self-esteem, a focused investigation of ingrained colorism within Khmer culture could provide valuable insights into the relationship between colorism and ethnic pride in the community, distinct from self-esteem, which was the main focus of this research. This study explores the multifaceted nature of colorism among Khmer women, primarily those living in the United States and Cambodia, and represents one of the first to focus on Khmer women in colorism literature. By doing so, it strengthens the understanding of colorism across the globe and opens numerous avenues for future studies on this population.

## Data Availability

The original contributions presented in the study are included in the article/supplementary material. Further inquiries can be directed to the corresponding author.
